# Atypical Mycobacterial Infection after Abdominoplasty Overseas: A Case Report and Literature Review

**DOI:** 10.1155/2016/3642567

**Published:** 2016-12-27

**Authors:** Prabin Sharma, Laia Jimena Vazquez Guillamet, Goran Miljkovic

**Affiliations:** ^1^Department of Internal Medicine, Yale New Haven Health System, Bridgeport Hospital, Bridgeport, CT 06610, USA; ^2^Department of Infectious Diseases, Yale New Haven Health System, Bridgeport Hospital, Bridgeport, CT 06610, USA

## Abstract

Increasing number of medical tourists travel internationally for cosmetic procedures.* Lipotourism* is a form of medical tourism becoming popular among patients of developed countries due to the cost efficiency of cosmetic procedures when performed in developing nations. There is a paucity of data on quality, safety, and risks involved with these surgeries. Many cases of infections have been documented in patients following cosmetic surgeries in developing countries. We present a case of a 34-year-old female who underwent abdominoplasty in Dominican Republic that was complicated with development of multiple abdominal wall abscesses due to infection from rapidly growing mycobacteria (RGM). In the absence of clear treatment guidelines, she was treated with a combination of intermittent surgical drainage and prolonged antibiotic course. This case is of interest as more than one species of RGM was isolated from the same patient. Our case highlights the fact that identification of these organisms can be difficult requiring referral of samples to specialized laboratories and treatment duration can last several months, which is determined by clinical and microbiological response.

## 1. Introduction

Travel to developing nations for cheaper surgical procedures is getting popular in the form of medical tourism. Modernization of technology, medicine, and medical facilities in low-income countries, access to information via Internet, and efficient air travel are facilitating this phenomenon [1]. Majority of these surgical procedures are for cosmetic reasons and body fat removal; thus, this practice is often termed* lipotourism* [2]. Surgeries performed in these settings are often complicated with skin and surgical site infections. Legal protection of medical tourists is usually absent due to inadequate malpractice laws in the destination countries [1]. Rapidly growing mycobacteria (RGM) has been associated with skin and surgical site infections in patients returning from cosmetic surgical procedures overseas [3–5]. We present another case of such infection caused by* M. chelonae-abscessus* complex and* M. fortuitum *complex.

## 2. Case Presentation

A 34-year-old female with past medical history significant for abdominoplasty in Dominican Republic three weeks prior to presentation came to our hospital with complaints of redness and swelling in the epigastric region. She developed abdominal discomfort a few days after she left the hospital and this progressed slowly over the weeks. Pertinent negative history included absence of nausea, vomiting, diarrhea, fever, chills, chest pain, shortness of breath, or changes in bowel habits. She also denied recent illness, sick contacts, or exposures.

**Figure 1:**
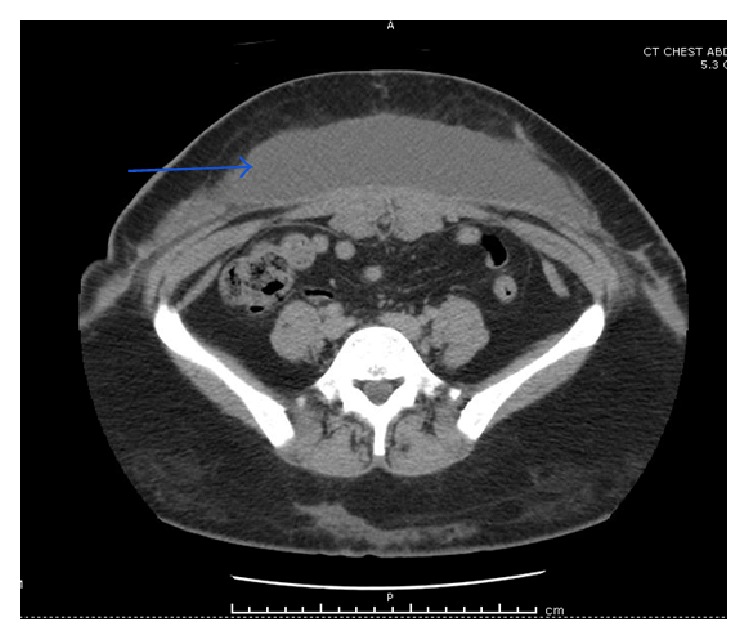
CT abdomen and pelvis with intravenous contrast showing large subcutaneous collection in the anterior abdominal wall (blue arrow) suggestive of abscess.

On arrival to the Emergency Department, her vitals were stable with blood pressure of 99/65 mm Hg, pulse rate of 100 beats per minute, and respiratory rate of 20 per minute. Her body temperature was 36.8°C and she saturated 98% in room air. Physical examination revealed an erythematous tender indurated area in the epigastrium approximately 2 cm in diameter. Complete blood count revealed a hemoglobin of 9.1 g/dl and white blood cell count of 13.7 × 10^9^ per liter (L) with 73% neutrophils. Both Erythrocyte Sedimentation Rate (ESR) and C-Reactive Protein (CRP) were elevated at 33 mm/hr and 2.3 mg/dL, respectively. Comprehensive metabolic panel was within normal limits. Computed Tomography (CT) of the abdomen and pelvis with contrast noted a large subcutaneous collection in the anterior abdominal wall suggestive of abdominal wall seroma (Figure 1). It also showed multiple areas of increased attenuation over the subcutaneous tissues of the abdomen, lower back, and bilateral flanks. Patient underwent CT guided drainage with placement of a drain in the anterior abdominal wall. During the procedure a sample of cloudy, straw colored fluid was sent for aerobic, anaerobic, and acid-fast bacilli (AFB) cultures. Ziehl-Neelsen stain was positive and rapid growth was detected in the AFB culture media. Samples were sent to Yale New Haven Hospital Laboratory for further identification. After 16 s rRNA sequencing analysis, the isolate was identified as* Mycobacterium fortuitum *complex. Patient was started on oral levofloxacin and doxycycline and discharged home with outpatient follow-up.

**Figure 2:**
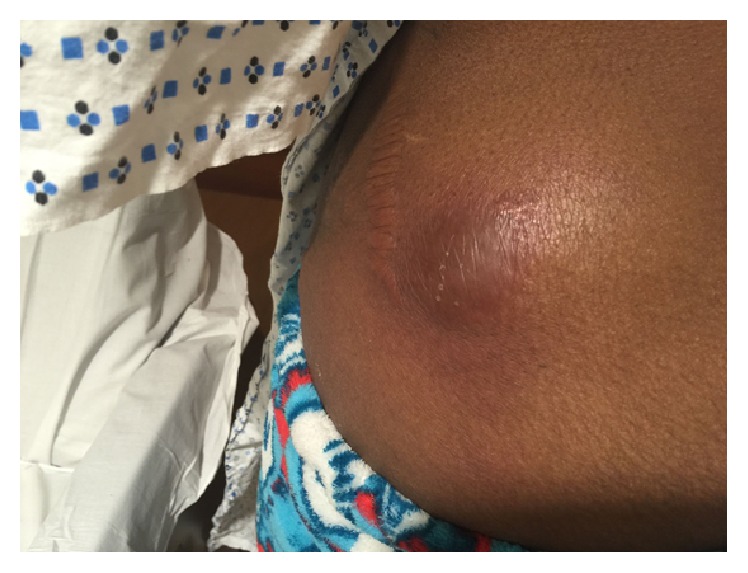
Soft, fluctuant, erythematous, and tender swelling in the lateral aspect of left hip.

**Figure 3:**
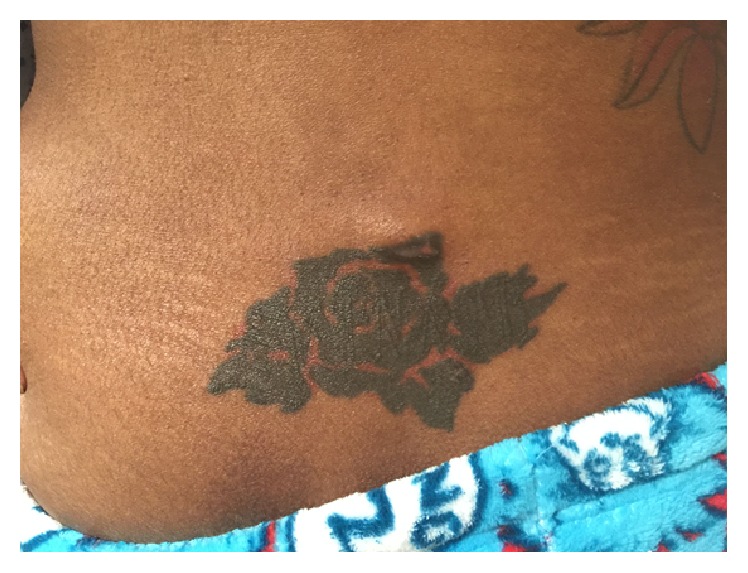
Soft, fluctuant, erythematous, and tender swelling in the lower central back (upper border of the tattoo).

**Figure 4:**
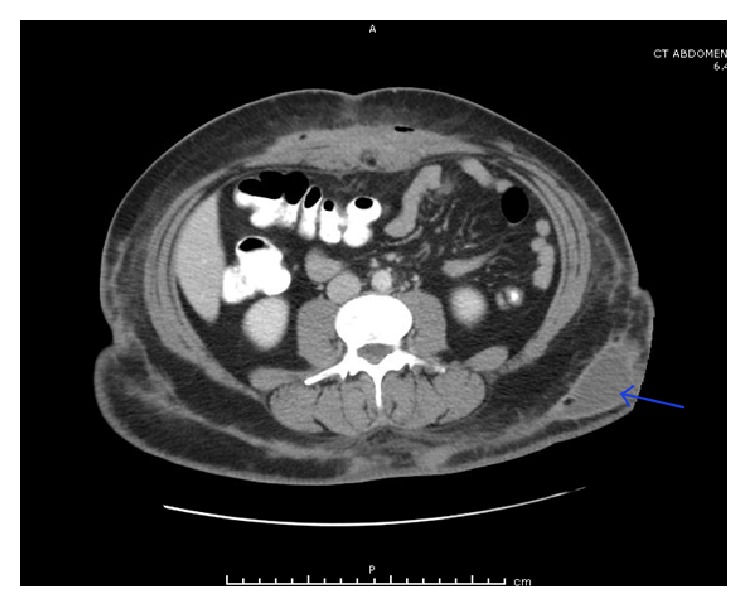
CT abdomen and pelvis with intravenous contrast showing subcutaneous rim-enhancing lesion in the left flank (blue arrow) suggestive of an abscess.

Two weeks later, she returned with two new soft tender swellings in her left lateral hip (Figure 2) and low mid back (Figure 3). A repeat CT abdomen and pelvis revealed significant resolution of the anterior abdominal wall fluid collection and subcutaneous rim-enhancing collection in the left flank suggestive of developing abscesses along with multiple other developing abscesses (Figure 4). She underwent incision and drainage and surgical debridement of the abdominal wall abscesses. Each abscess was incised at the pointed area with maximal threat of tissue breakdown. Incision was followed by immediate egress of pus under pressure. It was yellow, thick, and creamy, and a culture swab was taken of this for aerobic and anaerobic infection. Each wound was opened further and the wound cavity was cleaned by pulse irrigation. The wounds were then dressed using Iodoform gauze and ABD pads and secured with a Tegaderm with Mastisol used to keep the Tegaderm in place. She was started on empiric intravenous (IV) antibiotics (meropenem and amikacin) and moxifloxacin. Susceptibilities for* M. fortuitum *complex were ordered at the National Jewish Health Laboratory in Denver. New fluid samples collected during the second drainage were sent to the State of Connecticut's laboratory for identification.

After two weeks of intravenous antibiotics, IV amikacin was discontinued to avoid its adverse effects including interstitial nephritis, renal tubular necrosis, and ototoxicity. Doxycycline was added to the treatment with moxifloxacin and intravenous meropenem. The National Jewish Health laboratory identified* Mycobacterium senegalense* (one of the species that belongs to* Mycobacterium fortuitum *complex) by rpoB gene sequencing. The Mycobacterium was sensitive to amikacin, kanamycin, cefoxitin, imipenem, ciprofloxacin, doxycycline, moxifloxacin, tigecycline, clarithromycin, and azithromycin. It was noted to be resistant to sulfamethoxazole-trimethoprim, augmentin, and linezolid. After a total of twenty-one days of IV antibiotics, patient was discharged home on clarithromycin, doxycycline, and moxifloxacin. Final cultures from the second debridement identified* M. chelonae-abscessus group *using chemotaxonomic testing (high performance liquid chromatography (HPLC)); susceptibilities were not obtained. Patient has been on a regular follow-up with the infectious disease physician for seven months with slow progressive resolution of abdominal lesions.

## 3. Discussion

International travel in search of affordable health care or medical tourism is becoming very popular. Our patient travelled from United States to Dominican Republic with the aim of getting a cosmetic procedure for body fat removal.* Lipotourism* is a terminology used to describe practice of such travel for cosmetic surgeries for removal of body fat [5]. This practice has its financial benefits at the cost of other untoward risks. Data on quality, safety, and risks involved with these surgeries is lacking. Infections with RGM have been reported in cosmetic surgeries performed in the developing nations including Latin America and Caribbean. Increasing number of cases are also reported in Europe due to lipotourism to Eastern and Southern Europe [1, 2]. Most of the prior reports have found* M. abscessus* to be responsible for these infections.

Nontuberculous Mycobacteria (NTM) or atypical mycobacteria encompass a group of acid-fast organisms of mycobacteria species apart from* Mycobacterium tuberculosis* and* Mycobacterium leprae*. With advance in isolation and identification methods, there have been increasing incidence and reports of infections caused by this bacterial species ranging from skin and soft tissue infections to pulmonary and disseminated disease. Depending on the growth rate, NTM can be classified into rapidly growing mycobacteria (RGM) and slowly growing mycobacteria (SGM). RGM obtain their name from rapid growth in culture media within seven days. This group is subdivided into six different complexes based on genetic relatedness and pigmentation:* Mycobacterium fortuitum, Mycobacterium chelonae/abscessus, Mycobacterium mucogenicum, Mycobacterium smegmatis, early pigmenting RGM, and nonpigmented RGM* [6, 7].

*Mycobacterium fortuitum, Mycobacterium chelonae,* and* Mycobacterium abscessus* are three species of RGM responsible for the majority of clinical cases of skin and soft tissue infections in the United States [6]. Patients affected by* M. chelonae* usually have a predisposing immune suppression, while patients affected by* M. fortuitum* are immunocompetent patients that have suffered a trauma or an open laceration.* M. abscessus* can affect both normal hosts and immunocompromised patients [7]. Clinical findings of RGM are usually nonspecific and variable. Because of this variability in presentation, a high index of suspicion is required to diagnose skin infections caused by RGM.* M. chelonae* and M. abscessus usually present with multiple skin lesions. Severe and disseminated cutaneous disease is most common with* M. chelonae*. Classic presentation of skin infection caused by* M. fortuitum* is a single subcutaneous nodule at the site of trauma or surgery [6].

NTM are environmental bacteria that are found in natural and treated waters, soils, aerosols, and animals. Biofilms protect these mycobacteria from eradication by ordinary disinfection processes [6]. NTM are responsible for a wide array of infections, ranging from skin and soft tissue infections to osteomyelitis, pulmonary infections, and disseminated disease. Humans get infected by exposure to environmental reservoirs [8]. Skin and soft tissue infections result from colonization of mycobacteria by direct inoculation acquired via trauma, surgery, drug injections, and animal bites [3, 4, 6]. We believe that our patient was infected during her cosmetic procedure, since it has been shown that water supplies in hospitals can act as a reservoir leading to contamination of surgical instruments, irrigation solutions, and injectable medicines [6].

Diagnosis is established by biopsy of the skin lesions and abscess followed by cultures and histopathology. Gram stains and regular cultures usually do not yield any results and specific stains and cultures for mycobacteria are required [6, 9, 10]. RGM are specially sensitive to decontamination and discoloration procedures; therefore, it is difficult to rule out RGM infection with a negative smear [8]. Our patient had two different species of RGM isolated from different body locations, collected weeks apart. These varying results may reflect a true infection by different pathogenic RGM or simply an error in identification. We believe that she had a true infection and she was colonized by both bacteria species during her surgery in Dominican Republic. Our theory is further supported by the fact that the second set of cultures was obtained from lesions that were already developing on the first abdominal imaging. Also, the absence of any prior surgical soft tissue infections by RGM at our institution makes us less suspicious about an acquired infection while at our facility. Thus, this case reports coinfection by different rapidly growing mycobacteria after abdominoplasty. However, we want to acknowledge the possibility of an identification error since different techniques were used and HPLC has more limitations compared to molecular sequencing (discussion about identification techniques and its limitations is beyond the purpose of this article) [8].

Treatment usually requires a multidisciplinary approach and includes a combination of antibiotics with surgical drainage of abscess. There is no consensus in treatment and there are no prior well-controlled trials to guide treatment. RGM are susceptible to oral antibiotics: macrolides (clarithromycin, azithromycin), fluoroquinolones (ciprofloxacin, levofloxacin, and moxifloxacin), tetracyclines (doxycycline, minocycline), linezolid, and trimethoprim-sulfamethoxazole [10–12].* M. fortuitum* is more susceptible to drugs than* M. chelonae* or* M. abscessus*. Due to emergence of resistance and failure of treatment, monotherapy with a single agent is not recommended. Options for parenteral therapy include amikacin, imipenem, and levofloxacin [13, 14]. Treatment duration and route of administration are variable depending on site, severity of infection, and microbiological and clinical response. It is important to monitor drug toxicity while administering these antibiotics with regular follow-up of liver function, renal panel, and assessment of auditory and vestibular function [10]. Our patient has completed seven months of oral antibiotics with good response.

In summary, we present a case of a 34-year-old woman who acquired a soft tissue infection caused by RGM after an abdominoplasty overseas. In medical tourists presenting with infections following cosmetic surgery performed overseas, RGM should always be included in microbiological workup. Factors pointing towards the diagnosis of RGM include lack of response to conventional antibiotic regimen, recurrent wound infections, wound dehiscence, and poor wound healing as seen in our patient. Identification of these organisms can be difficult and referral of samples to specialized laboratories may be needed. Even though there are no clear guidelines, our patient responded well to a combination of antibiotic treatment and surgical debridement. Treatment can last several months and must be determined by clinical and microbiological response. What is unique about our case is the isolation of two different RGM species, which could have presented a treatment challenge in terms of antibiotic sensitivities. With this case, it is also important to raise awareness among physicians as well as general public about the risks involved with medical procedures performed in developing countries to avoid these complications.
